# A Vacuolar Invertase CsVI2 Regulates Sucrose Metabolism and Increases Drought Tolerance in *Cucumis sativus* L.

**DOI:** 10.3390/ijms23010176

**Published:** 2021-12-24

**Authors:** Lin Chen, Fenghua Zheng, Zili Feng, Yue Li, Muxuan Ma, Guoping Wang, Hongbo Zhao

**Affiliations:** 1 College of Horticulture, South China Agricultural University, Guangzhou 510642, China; chenlin_304@scau.edu.cn (L.C.); 20192017013@stu.scau.edu.cn (F.Z.); 930474894@stu.scau.edu.cn (Y.L.); chen07041@126.com (M.M.); 2 School of Bioscience and Engineering, Shaanxi University of Technology, Hanzhong 732001, China; fengzili2008@163.com

**Keywords:** *Cucumis sativus* L., vacuolar invertase, sucrose, drought tolerance

## Abstract

Vacuolar invertase (VI) can irreversibly degrade sucrose into glucose and fructose and involve in plants abiotic-stress-tolerance. Cucumber (*Cucumis sativus* L.) is susceptible to drought stress, especially during the seedling stage. To date, the involvement of VI in drought tolerance in cucumber seedlings is in urgent need of exploration. In the present study, a cucumber vacuolar invertase gene, *CsVI2*, was isolated and functionally characterized. The results showed that (1) CsVI2 showed vacuolar invertase activity both in vivo and in vitro; (2) the transcript level of *CsVI2*, along with VI activity, was significantly induced by drought stress. Moreover, the expression of sucrose synthase 3 (*CsSUS3*) was increased and that of sucrose phosphate synthase 1 (*CsSPS1*) was decreased after exposure to drought stress, which was followed by an increase in sucrose synthase activity and a decrease in sucrose phosphate synthase activity; (3) *CsVI2*-overexpressing transformed cucumber seedlings showed enhanced vacuolar invertase activity and drought tolerance and 4) protein–protein interaction modelling indicated that a cucumber invertase inhibitor, CsINVINH3, can interact with CsVI2. In summary, the results indicate that CsVI2 as an invertase can regulate sucrose metabolism and enhance drought stress in cucumber seedlings.

## 1. Introduction

Sucrose metabolism plays important roles in plant development, yield formation and stress response [1]. In most plants, sucrose is the end product of photosynthesis and is the major form of carbon that is transported from the source, mainly from leaves to sinks, such as fruit and roots [2]. Sucrose is further enzymatically degraded into glucose and fructose to provide energy and carbon sources for various metabolic processes [1,2]. In higher plants, sucrose hydrolysis was conducted by sucrose synthase (SUS) and invertase. SUS catalyzes the reversible reaction that degrades sucrose into fructose and uridine diphosphate glucose [1,3,4]. Invertase is a key enzyme in sucrose hydrolysis that can hydrolyze sucrose into glucose and fructose [5], plays vital roles in providing carbon nutrients to plants and plays major roles in sugar signaling and tissue development [1,2].

In plants, invertases are classified into two types based on their optimum potential for hydrogen: neutral/alkaline invertases and acid invertases [6]. Acid invertases localize in either the cell wall as tightly bound forms or in the vacuole as soluble forms [7]. Vacuole invertase (VI) is a key modulator of plant hexose accumulation [2]. In many plants, high VI activity usually correlates with more hexose accumulation [2,8,9]. Since terrestrial plants are sessile organisms and are unable to move, they have developed a flexible sucrose metabolism to accommodate environmental changes. Sucrose metabolism aids growth and development under optimum conditions and shifts to abiotic stress tolerance in adverse environments. In addition to the roles in primary metabolism and plant development mentioned above, acid invertase, which mediates sucrose metabolism, plays an important role in abiotic stress [1], such as salinity [10] and drought [11].

Drought, one of the most detrimental abiotic stresses, has a severe effect on plant yield losses [12]. With global warming, drought often occurs simultaneously with heat and leads to a synergistic adverse effect on crop productivity under field conditions [13]. Thus, increasing the drought tolerance of crops is an essential option for sustaining crop yield. Drought stress influences plant growth, adaptive responses and crop yield by modifying the source-sink relation [14]. Since sucrose metabolism determines the source-sink relationship, it is vital to know how drought stress affects sucrose metabolism and, more importantly, how plants change invertase activity to increase drought stress tolerance. Transgenic tomato lines with ectopic overexpression of the cell wall invertase *CIN1* showed enhanced drought tolerance [14]. It is implied that cell wall invertase can contribute to drought tolerance. However, whether VI can participate in drought tolerance is not yet completely understood.

Cucumber (*Cucumis sativus* L.), originating from the southern Himalayas, is an important horticultural crop and is sensitivity to water deficiency [15,16]. Like most cucurbitaceous plants, cucumber has a high transpiration rate and is not tolerant of drought stress [17]. High drought stress will have obvious negative effects on the growth, photosynthesis, biochemistry, quality and yield of cucumber fruits [18,19]. Especially during the seed germination and seedling stages, cucumber is extremely vulnerable to drought stress [20].

To date, whether VI is involved in drought tolerance in cucumber is still unknown. In this study, a previously uncharacterized vacuolar invertase in cucumber, *CsVI2*, was functionally characterized. In addition, whether *CsVI2* plays important roles in cucumber related sucrose metabolism and drought tolerance was explored. The results indicated that the CsVI2 protein has vacuolar invertase activity both in vivo and in vitro. Expression of *CsVI2* was induced by drought. Transgenic cucumber seedlings with *CsVI2* overexpression showed enhanced VI activity and drought tolerance. Overall, this study revealed that CsVI2 can regulate sucrose metabolism and increase drought tolerance in cucumber.

## 2. Results

### 2.1. Cloning and Enzymatic Characterization of CsVI2

Previously, three cucumber putative vacuolar invertase genes have been screened, and only vacuolar invertase *CsVI1* was functionally characterized by us [21]. In this study, *CsVI2* (Csa5G1704590.1), was chosen for further research. The CDS length of *CsVI2* was 1893 bp, and its predicted protein molecular weight was 69.68 kDa. Additionally, the predicted isoelectric point of the *CsVI2* protein was 4.69, which suggests that it can be an acid invertase-like protein. Signal peptide prediction by PSORT (http://psort.hgc.jp/, accessed on 10 May 2019), Target P (http://www.cbs.dtu.dk/services/TargetP/, accessed on 10 May 2019) and SIGNAL P (http://www.cbs.dtu.dk/Services/SignalP/, accessed on 10 May 2019) indicated that *CsVI2* is localized in the vacuole. In addition, *CsVI2* is located on chromosome five according to its chromosomal annotation.

**Figure 1:**
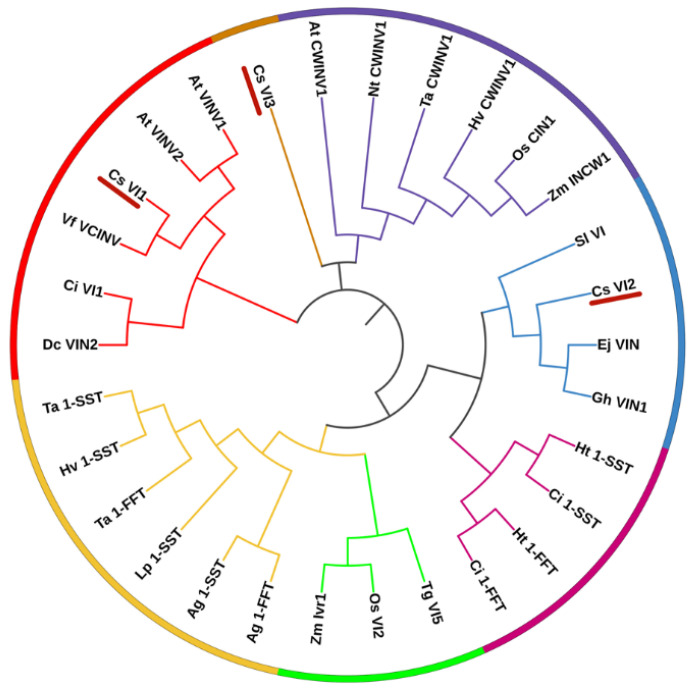
Phylogenetic analysis of vacuolar invertases and vacuolar invertase-like amino acid sequences. Dicot vacuolar invertases are marked with red and blue curves. Cell wall invertases are marked with purple curves. Monocot FTT and SST are marked with yellow curves. Dicot FFT and SST are marked with burgundy curves. Monocot VIs are marked with green curves. CsVI3 is marked with orange curve. CsVI1 to 3 are underlined with red line. Species names are abbreviated as Ag, *Agave tequilana*; At, *Arabidopsis thaliana*; Ci, *Cichorium intybus*; Cs, *Cucumis sativus*; Dc, *Daucus carota*; Ej, *Eriobotrva japonica*; Gh, *Gossypium hirsutum*; Ht, *Helianthus tuberosus*; Hv, *Hordeum vulgare*; Lp, *Lolium perenne*; Nt, *Nicotiana tabacum*; Os, *Oryza sativa*; Sl, *Solanum lycopersicum*; Ta, *Triticum aestivum*; Tg, *Tulipa gesneriana*; Vf, *Vicia faba var. minor*; Zm, *Zea mays*.

Phylogenetic analysis was further carried out on the amino acid sequences of *CsVI2*, other *VIs* and *VI*-like genes from different species. The results showed that the genes were classified into seven groups (Figure 1). *CsVI2* was not classified into CWI but was grouped into VI (Figure 1). Additionally, *CsVI2* was grouped with dicot VI from *Eriobotrva japonica*, *Solanum lycopersicum* and *Gossypium hirsutum* (Figure 1).

As VI is highly similar with fructosyltransferase in the evolution to exclude CsVI2 as a fructosyltransferase, and the phylogenetic comparison was further analysed with some fructosyltransferases added, including 1-SST (sucrose: sucrose 1-fructosyltransferase) and 1-FFT (fructan: fructan 1-fructosyltransferase). The results showed that there was an obvious difference between CsVI2 and fructosyltransferase (Figure 1). It suggested that CsVI2 is not a member of the fructosyltransferase family.

**Figure 2:**
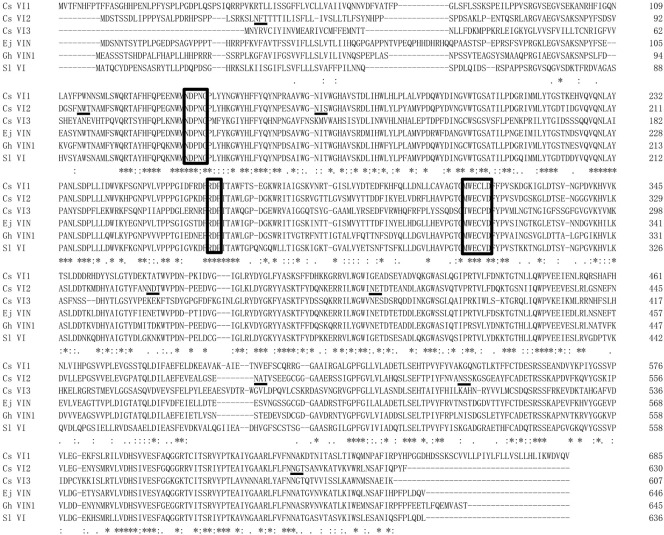
Amino acid sequence alignment of CsVIs and vacuolar invertase from other dicot species. β-fructosidase motifs (N**D**PD/NG), cysteine-containing catalytic sites (WEC**V**D), and R**D**P are boxed. Putative glycosylation sites are underlined with black lines. Asterisks indicate identical residues, colons indicate conserved substitutions, and periods indicate semiconserved substitutions. The scheme was derived from Clustal Omega (https://www.ebi.ac.uk/Tools/msa/clustalo/, accessed on 2 June 2019).

Conserved motif and residue analyses were further performed on the amino acid sequences of CsVIs and VI from *Eriobotrva japonica*, *Solanum lycopersicum* and *Gossypium hirsutum*. CsVI2 has three conserved motifs consisting of the β-fructase motif N**D**PD/NG, cysteine-containing catalytic site WEC**V**D and R**D**P (Figure 2). Moreover, eight predicted glycosylation sites were predicted in the amino acid sequence (Figure 2).

**Figure 3:**
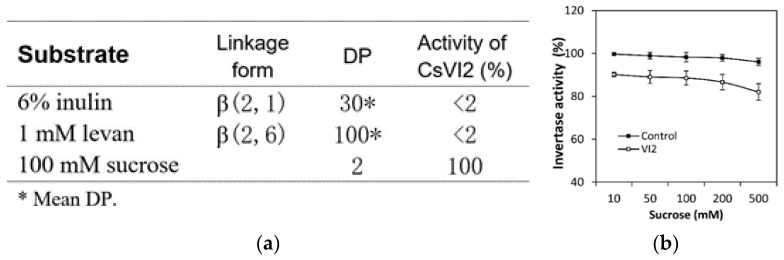
Enzymatic activity analysis of CsVI2 recombinant protein from *Picha pastoris*. Substrate specificity analysis (**a**) and enzyme activity comparison (**b**) between recombinant CsVI2 and commercial invertase. The results are the means of at least three biological replicates (±S.E.), each with three technical replicates.

**Figure 4:**
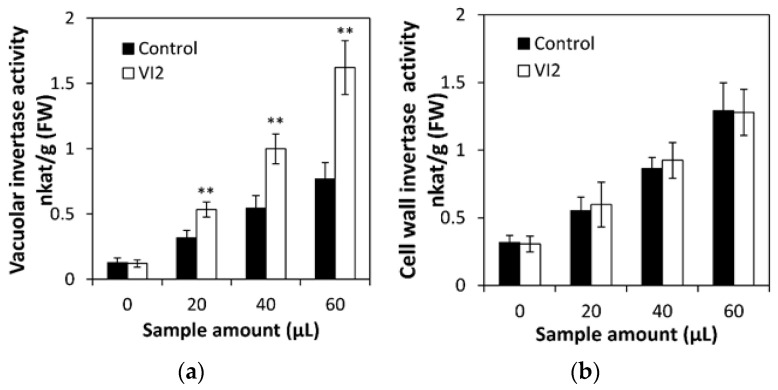
Vacuolar- and cell wall-associated invertase activities in *Nicotiana benthamiana* leaves by transient transformation with *CsVI2*. Substrate concentrations consisted of 100 mM sucrose. The invertase activity induced by transformation with empty vector alone was subtracted. The results are the mean of four biological replicates (±S.E.), each with three technical replicates. Asterisks indicate statistically significant differences by using Student’s *t* test (**, *p* value < 0.01).

To identify the enzymatic specificity of CsVI2, first, an in vitro system was used for the heterologous expression of the CsVI2 recombinant protein by *Picha pastoris*. A recombinant CsVI2 protein was used, and the enzyme activity was quantified to test the content of released fructose and glucose from the substrate sucrose. The results showed that recombinant CsVI2 protein can hydrolyze sucrose into glucose and fructose after coincubation with sucrose with almost no affinity to other sugars—inulin and levan (Figure 3a). Additionally, compared with a commercial acid invertase, the recombinant CsVI2 protein has a similar trend in sucrose hydrolyzation followed by an increased sucrose concentration (Figure 3b). To identify the enzymatic specificity in vivo, a transient expression system for CsVI2 protein in tobacco leaves was conducted. The results showed that overexpressed *CsVI2* elevated invertase activity specifically in the vacuole (Figure 4a). In addition, invertase activity in the cell wall did not have a distinct variation compared with the control (Figure 4b). Briefly, these results demonstrate that CsVI2 is a vacuolar invertase that can degrade sucrose in vivo and in vitro.

### 2.2. Expression Profile of CsVI2 in Cucumber Seedlings

**Figure 5:**
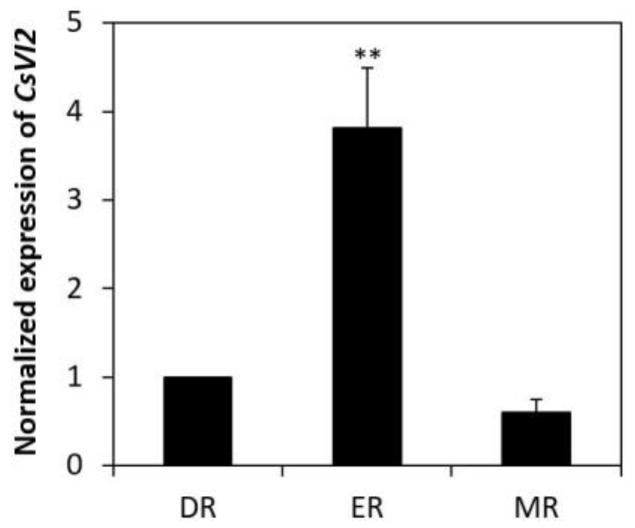
Tissue-specific expression of *CsVI2* in cucumber seedlings. Expression of *CsVI2* in different root parts. DR, differentiated root region (maturation region); ER, elongation root region; MR, meristematic root region. The results are the mean of at least three biological replicates (±S.E.), each with three technical replicates. Asterisks indicate statistically significant differences by using Student’s *t* test (**, *p* value < 0.01).

The expression of *CsVI2* was detectable in the leaves, roots and stems, and especially peaked in young leaves and young roots, according to our previous research [21]. To further understand the expressional parts of *CsVI2* in young roots, young cucumber roots were divided into maturation regions, elongation regions and meristematic regions. The results showed that *CsVI2* was expressed in all of the regions and had the highest expression level in the elongated part (Figure 5).

### 2.3. Drought Stress Induces CsVI2 Expression and Increases Vacuolar Invertase Activity

**Figure 6:**
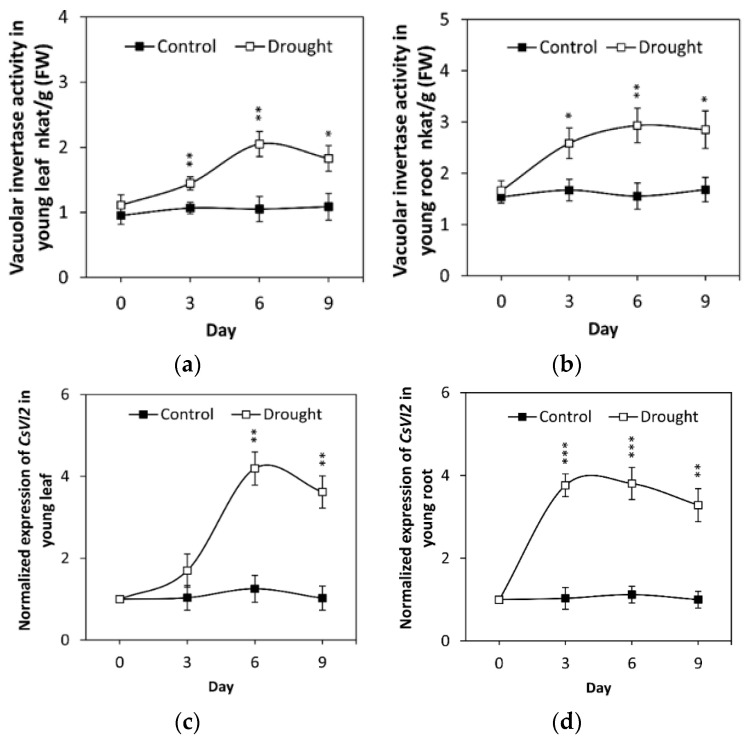
Drought stress affects vacuolar invertase activity and *CsVI2* expression in young leaves and young roots of cucumber seedlings. The results are the means of three biological replicates (±S.E.), each with three technical replicates. Asterisks indicate statistically significant differences by using Student’s *t* test (*, *p* value < 0.05; **, *p* value < 0.01; ***, *p* value < 0.001).

To evaluate the effect of drought on cucumber VI, the cucumber seedlings were treated with drought stress. Samples were collected from 0 to 9 days after treatment, and the vacuolar invertase activities were measured from young leaves and young roots. The results indicated that vacuolar invertase activities were significantly induced in both tissues from the 3rd day after drought (Figure 6a,b). Moreover, the transcriptional regulation of *CsVI2* under drought stress conditions was also assayed. The expression of CsVI2 in young roots increased significantly after 3 days of drought treatment, while a trend of a significant increase was found in young leaves from the 6th day (Figure 6c,d). These data indicated that drought induces cucumber vacuolar invertase activity and VI2 expression in the whole seedling.

### 2.4. Drought Stress Effects SUS and SPS Activity and Related Gene Expression

**Figure 7:**
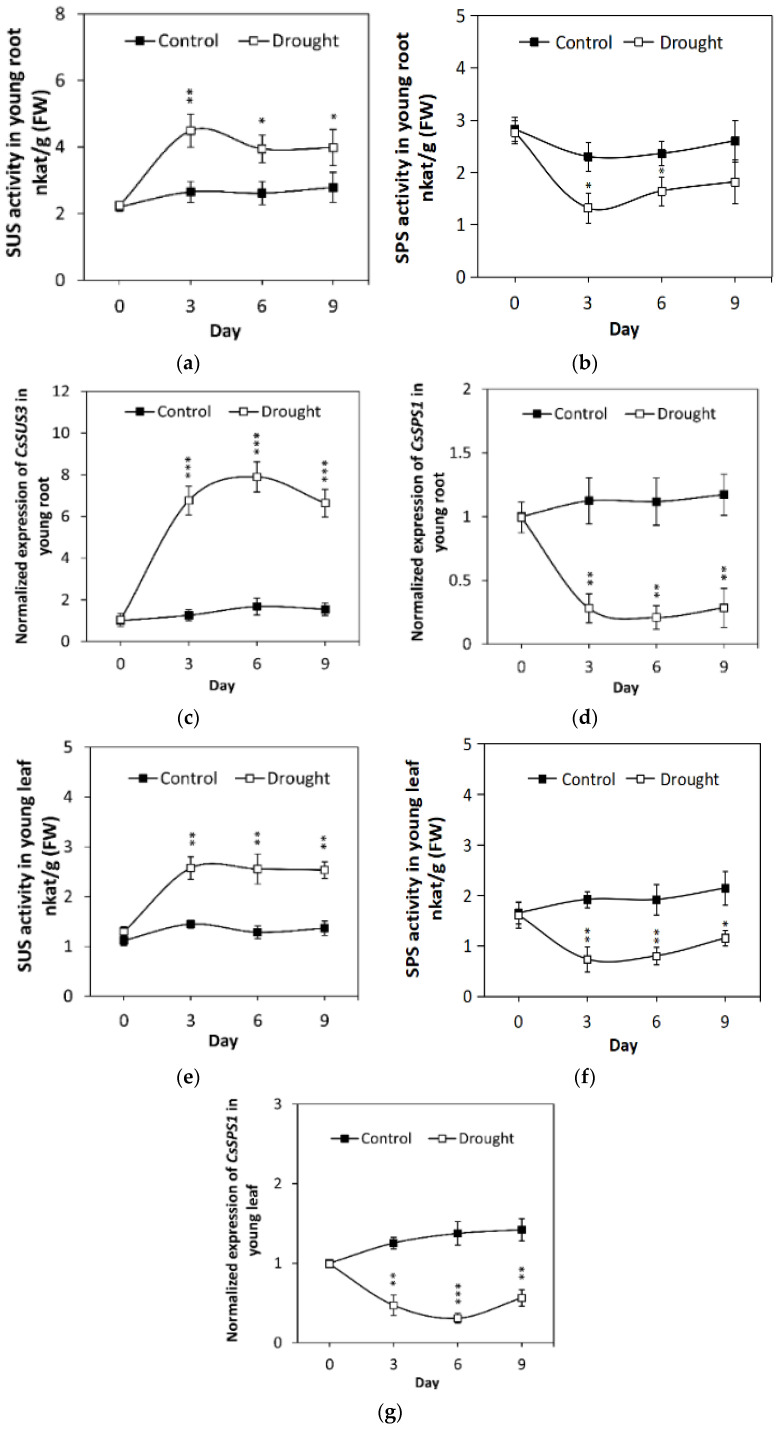
Drought stress effects *CsSUS3* and *CsSPS1* expression and their enzyme activities in young leaves and young roots of cucumber seedlings. The results are the means of at least three biological replicates (±S.E.), each with three technical replicates. Asterisks indicate statistically significant differences found by using Student’s *t* test (*, *p* value < 0.05; **, *p* value < 0.01; ***, *p* value < 0.001).

To further understand the effects of drought on cucumber sucrose metabolism, the activities of sucrose synthase (SUS) and sucrose phosphate synthase (SPS) were assayed. For SUS, the enzymatic activities of young roots and young leaves in cucumber seedlings were increased after exposure to drought (Figure 7a,e). SUS activity peaked on the 3rd day of drought treatment (Figure 7a,e).

Previous studies have found four putative cucumber *SUS* genes and one *SPS* gene in cucumber [22,23]. To uncover *SUS-* and *SPS*-related gene expression variation in cucumber under drought stress, as we found that only *CsSUS3* was highly expressed in the roots, and *CsSPS1* was highly expressed in roots and leaves, so *CsSUS3* and *CsSPS1* were selected. Gene expression analysis indicated that a root-specific sucrose synthase gene, *CsSUS3*, was significantly upregulated after drought treatment (Figure 7c). As the cucumber leaf-targeted sucrose synthase gene has not been found thus far, we speculated that there should be a novel sucrose synthase gene responsible for drought-induced SUS activity in the young leaves of cucumber seedlings. For SPS, its enzymatic activities in young roots and young leaves in cucumber seedlings were decreased by drought, especially on the 3rd day (Figure 7b,f). Gene expression analysis indicated that *SPS1* was downregulated sharply in both the young root and young leaf tissues from cucumber seedlings (Figure 7d,g).

### 2.5. Overexpressing CsVI2 Enhanced Vacuolar Invertase Activity and Drought Tolerance in Cucumber Seedlings

**Figure 8:**
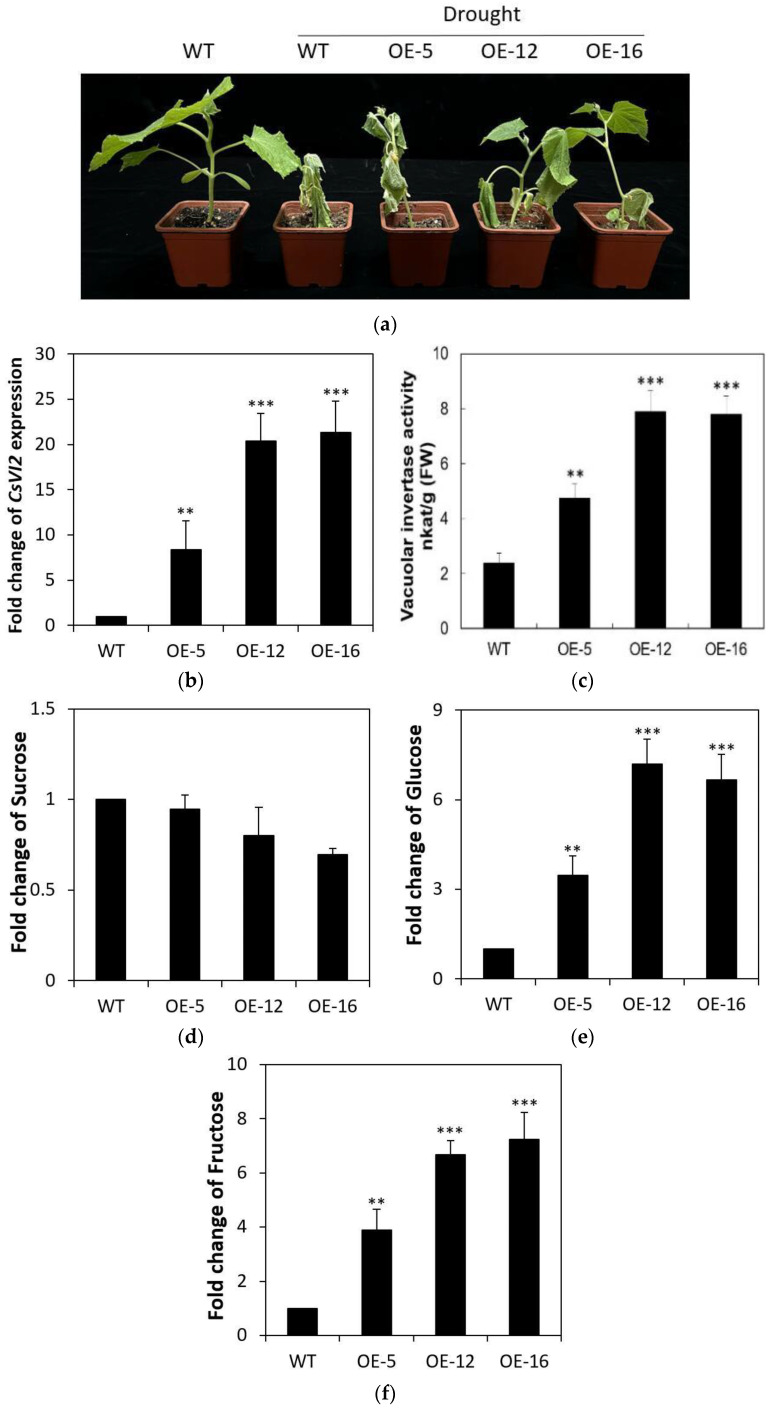
Drought-treated phenotypes, expression of CsVI2, vacuolar invertase activity, concentration of sucrose and hexoses of WT and CsVI2-overexpressing transgenic lines. (**a**) Phenotype of WT and CsVI2-overexpressing transgenic lines after drought treatment for two weeks. (**b**) Expression of CsVI2 in leaves of WT and CsVI2-overexpressing transgenic lines. Vacuolar invertase activities (**c**) and concentrations of sucrose (**d**), glucose (**e**) and fructose (**f**) in leaves of WT and CsVI2-overexpressing transgenic lines. The results are the means of more than three biological replicates (±S.E.), each with three technical replicates. Asterisks indicate statistically significant differences by using Student’s *t* test (**, *p* value < 0.01; ***, *p* value < 0.001).

To investigate the role of CsVI2 in the drought tolerance of cucumber, *CsVI2*-overexpressing transgenic lines were obtained and three lines (5, 12 and 16) were used for further identification. After drought treatment for two weeks, these three transgenic lines showed enhanced drought tolerance compared with the wild type (Figure 8a). In parallel with the enhanced drought tolerance in cucumber seedling leaves, especially for OE-12 and OE-16, they showed significantly increase in both *CsVI2* expression and vacuolar invertase activities (Figure 8b,c). Additionally, the lines showed mildly decreased sucrose accumulation and significantly increased hexose contents in cucumber seedling leaves, especially for OE-12 and OE-16 (Figure 8d–f).

### 2.6. CsVI2 can Form a Complex with a Putative Cucumber Invertase Inhibitor CsINVINH3

Invertase activity is post-translationally inhibited by invertase inhibitor [24]. Up till now, little information on the cucumber invertase inhibitor has been known. To reveal the possible interaction between CsVI2 and the cucumber invertase inhibitor, a putative cucumber invertase inhibitor, *CsINVINH3* (Csa.302150), was used for modelling protein–protein interaction.

**Figure 9:**
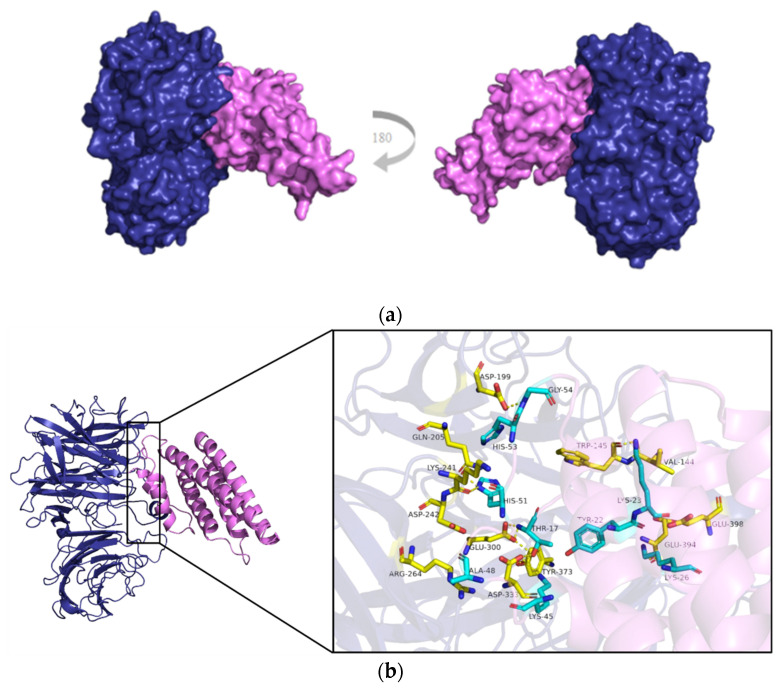
Proposed structure modelling of CsVI2—CsINVINH3 complex. (**a**) Overview of CsVI2 (purple)—CsINVINH3 (Purplish red) complex structure. (**b**) Proposed interacting interface between CsVI2 and CsINVINH3. comprised of conserved amino acids or motifs of the modelled interacting proteins (right). Amino acids with blue are from CsINVINH3, and amino acids with yellow are from CsVI2. Hydrogen bonds are marked as dotted lines.

The AtCWI1—NtCIF complex structure [25] was used as a template for modelling the CsVI2–CsINVINH3 interaction. As can be seen, CsINVINH3 indeed binds to the active sites of CsVI2 and may exert its inhibitory effect on CsVI2 activity (Figure 9a,b). Try-17 in CsINVINH3 combines three amino acids (Gly300, Asp333 and Try373) in CsVI2, and Lys45 in CsINVINH3 also acts on Asp333 in CsVI2 (Figure 9b). In addition, Tyr22, Lys26, His53 and Gly54 in CsINVINH3 were found can combine Glu398, Glu394, Gln205 and Asp199 in CsVI2 (Figure 9b). For Ala48 and His51 in CsINVINH3 can combine with Asp242, Arg264, Gln205 and Lys241, respectively (Figure 9b). In CsINVINH3, except for Tyr22, Lys23 and Lys45 are located in the first α-helix, and all other binding amino acids are targeted to the linear region of CsINVINH3 (Figure 9b).

## 3. Discussion

Expression of *CsVI2* and VI activity were increased after exposure to drought stress in cucumber seedlings (Figure 6). Besides *CsVI2*, drought stress did not induce the *CsVI1* expression in the transcript, and *CsVI3* has been found almost no expression of in young cucumber seedling tissues [21]. The similar phenomenon was also found in maize [5], rice [26] and Arabidopsis [11]. Those findings indicate that the expression pattern of Cs*VI2* in parallel with VI activity is highly related to drought stress, and *CsVI1* or *CsVI3* is not the key VI which specifically response to the drought stress. Additional transgenic work showed that *CsVI2* overexpression lines have an increase in drought stress tolerance (Figure 8). This implied that drought-responsive *CsVI2* is a process by which plants increase drought tolerance.

Drought stress can promote both sucrose and hexose accumulation in plants [27]. Sucrose and hexose can help cells to prevent dehydration under drought conditions [28]. Drought stress, accompanied by deficiency of the water supply, inhibits cucumber plant growth. Osmotic pressure is thought to play a major role in cell expansion and plant growth [1]. Chen et al. (2016) indicated that the activity of VI is correlated with the stomatal aperture, in which the rapid closure of the stomata contributes to drought tolerance [11]. As CsVI2 promotes sucrose conversion to glucose and fructose, which doubles the osmotic contribution of sucrose, the increase in *CsVI2* expression in parallel with VI activity reduce the inhibition of plant growth by drought.

*CsVI2* is mainly expressed in the elongation region of cucumber seedling roots (Figure 5). Previously, *AtVI2* from *Arabidopsis thaliana* was proved to be able to regulate root elongation [14], and crops with large and deep root systems can absorb more water from the soil to enhance drought stress tolerance [11]. As the elongation region of the root is where newly formed cell length increases, it leads to root lengthening. It is speculated that *CsVI2* may be also involved in cucumber root development, and speculated that *CsVI2* related VI activity might improve drought stress tolerance by improving root length.

In addition to its potential root-promoting strategy, *CsVI2* might also improve drought tolerance by reducing the content of reactive oxygen species. Drought stress often leads to deleterious levels of reactive oxygen species, which cause oxidative damage to cellular membranes, proteins, DNA and RNA and, finally, cause programmed cell death [25]. Glucose can feed on the oxidative pentose phosphate pathway and participate in the biosynthesis of antioxidants, such as ascorbic acid and glutathione, by producing reducing power. The product antioxidants can scavenge reactive oxygen species efficiently [13,25]. Sinkevich et al. showed that potato transgenic lines overexpressing yeast invertase showed improved cold tolerance by enhancing the antioxidant capability [29].

Drought stress may regulate the overall sucrose metabolism cycle in cucumber. Drought induced the up-regulation of *VI* genes and also was accompanied with the variations of *SUS* and/or *SPS* related genes [30,31,32,33,34]. Except VI, sucrose is also degraded by SUS; it catalyzes sucrose and UDP into fructose and UDP-glucose [2,35]. The regulation of SUS in cucumber may also connect to drought response (Figure 7). SPS is a key regulator of sucrose synthesis and is responsible for sucrose synthesis in plants [2,22,36]. Till now, how SPS was regulated by drought remains controversial. In rice, drought stress was reported that increased SPS activity in leaves and stems [30]. However, our results in cucumber seedlings found that drought decreased the SPS activity and *SPS1* expression (Figure 7). One latest example supported our result was that drought stress increased SPS activity and *GmSPS* expression in soybean [33].

In cucumber, sucrose was synthesized from source leaves by photosynthates, and loaded into the phloem to remobilize to the sink tissues, such as the young leaf and young root of seedlings. Drought can increase the seedlings VI activity by the up-regulation of *CsVI2* expression and induce more hexose accumulation. Meanwhile, *CsSUS3*-enhanced SUS activity also helped more hexose production, while SPS activity was decreased possibly by the down-regulation of *CsSPS1* under drought. In short, the variation of activity of SUS and SPS, combined with CsVI2, promotes the content of hexoses, which might improve cucumber drought tolerance by promoting growth and ROS scavenging.

Invertase inhibitor can post-translationally regulate the VI activity [24]. Modeling results showed that CsINVINH3 can tightly bind to the active sites of CsVI2 and may exert its inhibitory effect on CsVI2 and the substrate reaction (Figure 9), suggesting that CsINVINH3 might act as an invertase inhibitor to inhibit CsVI2 activity in cucumber. Whether CsINVINH3 regulates cucumber tolerance of drought needs further exploration.

## 4. Materials and Methods

### 4.1. Plant Material and Treatments

Cucumber (*Cucumis sativus* cultivar ‘HC-1’) and *Nicotiana benthamiana* L. were cultivated at 23 ± 2 °C under long-day conditions (16 h/8 h light period; 300 μmol m^−2^ s^−1^). Cucumber seedlings at the two-leaf stage were chosen for the water interruption treatment. Samples were collected at 0 day, 3 day, 6 day and 9 day. The collected tissue samples were either analysed immediately or shock-frozen in liquid nitrogen and stored at −80 °C prior to analysis. Tobacco leaves from 8- to 12-week-old plants were used for transient transformation. Leaf samples were collected after transient transformation for 48 h.

### 4.2. Gene Expression Analysis

Total RNA was extracted using the HiPure Plant RNA Kit (Magen, China). RNA samples were treated with DNase I (Takara, Japan), with treatment immediately followed by cDNA synthesis using AMV-Reverse Transcriptase (NEB, UK). Quantitative real-time PCR (qPCR) analysis was performed using the Rotor-Gene 3000 system (Corbett Research, Oatley, Australia) using SYBR Premix ExTaq II (TAKARA BIO, Shiga, Japan) to monitor dsDNA synthesis. Thermal cycling conditions were identical for all primer pairs: 95 °C/5 min, followed by 35 cycles of 95 °C/20 s–58 °C/20 s–72 °C/20 s, followed by a melt cycle from 50 to 95 °C. To determine primer efficiency, serial dilutions of the templates were conducted for all primer combinations. The expression level of the target gene was quantified with the reference gene tubulin. Each reaction was performed in four biological replicates. The relative expression level of the target gene was calculated by normalizing to the geometric mean of the reference genes. Primers for the reference gene and target gene are presented in Table S1 (available as Supplementary Material to this paper).

### 4.3. Plant Transformation

The coding region of *CsVI2* was cloned into the pB7WG2 vector under the control of the CaMV 35S promoter (primers shown in Table S1). Transient expression in tobacco leaves was performed by *Agrobacterium* leaf infiltration as previously reported [37]. The control was transformed with P19. Cucumber transformation was carried out by using the cotyledon transformation method as previously reported [38]. The positive transgenic plants were verified by PCR, and the expression of *CsVI2* in the transgenic plants was further assayed by qPCR.

### 4.4. Plant Carbohydrates and Protein Extraction

Total soluble carbohydrates from cucumber seedlings were extracted as described previously [39]. The vacuolar and cell wall-bound proteins from cucumber and *Nicotiana benthamiana* leaves were extracted as described in Su et al. [39]. Bounded proteins were eluted from the resuspended cell wall fraction with 500 mM NaCl for one hour at 4 °C, followed by centrifugation at 10,000× *g* at 4 °C. Soluble and salt-eluted cell wall proteins were washed and concentrated by a centrifugal filter (MilliporeSigma, Burlington, MA, USA) with 50 mM sodium acetate buffer (pH 5.0). The protein concentration was determined by Bradford assay.

### 4.5. Heterologous Expression and Purification of CsVI2

For the generation of the expression plasmid in *Pichia pastoris*, the coding region of *CsVI2* was inserted into the pPICZα vector (Invitrogen, Carlsbad, CA, USA) linearized with *Eco*RI and *Xba*I. The recombinant plasmid was transformed into *E. coli*-competent DH5α cells by electroporation and screened on zeocin plates. Positive colonies were used for vector amplification. Subsequently, the plasmid was extracted and linearized by *Pme*I and then transformed into *Pichia pastoris* strain X-33 via electroporation. Further selection and protein purification were performed as described by Kusch et al. [37].

### 4.6. Enzyme Activity and Carbohydrate Assay

Vacuolar invertase protein was incubated with 10–500 mM sucrose (Sangon Biotech, Shanghai, China) in 50 mm sodium acetate buffer (pH 5.0) at 37 °C for different time intervals. After incubation, the reaction was stopped by heating at 95 °C for 5 min. The contents of fructose and glucose were measured by a coupled spectrophotometric enzyme assay, as described previously [21]. All enzyme measurements were performed under conditions where activities were linearly proportional to the amount of enzyme and incubation time.

### 4.7. Protein Complex Modelling

Modelling of CsVI2 in a proposed complex with CsINVINH3 was based on the structure of the complex between AtCWI1 and the tobacco invertase inhibitor Nt-CIF [25] (PDB entry 2XQR, https://www.rcsb.org/, accessed on 15 September 2021). Structural alignments and model calculations were performed using Modeller 9.25 software [40]. Protein docking analysis was performed using Discovery Studio 2020 (Biovia) and visualized in Discovery Studio Visualizer 2020 (Biovia). High-resolution rendering was performed using PyMOL (https://pymol.org/2, accessed on 17 September 2021) to verify the binding ability of the modelled protein complex.

### 4.8. Statistical Analysis

Statistical analysis was performed using SPSS software (version 21.0, SPSS Institute, Armonk, NY, USA). Mean values from the treatments were compared using Student’s *t* test. Asterisks indicate significant differences (*, *p* value <0.05; **, *p* value < 0.01; ***, *p* value < 0.001).

## 5. Conclusions

In this study, the vacuolar invertase CsVI2 was functionally characterized, and the expression of *CsVI2* and related enzyme activity were significantly induced by drought stress. Moreover, drought increased sucrose synthase (SUS) activity but decreased sucrose phosphate synthase (SPS) activity, which was followed by the upregulation of *CsSUS3* and downregulation of *CsSPS1* expression. Finally, overexpression of *CsVI2* enhanced the vacuolar invertase activity and drought tolerance in cucumber seedlings. Taken together, the results indicate that CsVI2 is involved in sucrose metabolism and regulates drought stress in cucumber seedlings.

## Supplementary Materials

The following supporting information can be downloaded at: https://www.mdpi.com/article/10.3390/ijms23010176/s1.
