# Supplementary material for: A Vacuolar Invertase CsVI2 Regulates Sucrose Metabolism and Increases Drought Tolerance in Cucumis sativus L
Source: Int J Mol Sci. 2021 Dec 24;23(1):176. doi: 10.3390/ijms23010176 (PMC8745504; doi:10.3390/ijms23010176)
Supplement: Supplementary file 1 [file ijms-23-00176-s001.zip › ijms-1496269-supplementary.pdf]

Supplementary Materials:

Table S1. Primer list. Primers for gateway cloning, real-time PCR, and protein heterologous expression (pPICZ).

cDNA	Comment	Primer	Primer sequence (5′- 3′)
VI2	gateway	Forward	GGGGACAAGTTTGTACAAAAAAGCAGGCTCCATGGATTCAACTTCT TCAGAT
		Reverse	GGGGACCACTTTGTACAAGAAAGCTGGGTCTCAAAAATATGGTTGA ATGA
	pPICZ	Forward	AATCCGGAATTCTCTCATGGATTCAACTTCTTCAGA
		Reverse	GACTAGTCTAGACCAAAATATGGTTGAATGAAGG
	Real-time PCR	Forward	CTGGGCCTCTGTTCAGGTAA
		Reverse	CCCCAATCTCAAACTCTCCA
SPS1	Real-time PCR	Forward	ACACGTTTGCCAAGAATCAGTT
		Reverse	CAAGCCTTCTGAATTAGTTGGAGTC
SUS3	Real-time PCR	Forward	AGAGACCGAGAAAAGGCTAACT
		Reverse	AACTAACTCCCTAAGACGATCG
tubulin	Real-time PCR	Forward	ACGCTGTTGGTGGTGGTAC
		Reverse	GAGAGGGGTAAACAGTGAATC
